# Successful Treatment of Ventricular Arrhythmia in Tetralogy of Fallot Repair Using Catheter Ablation

**DOI:** 10.21470/1678-9741-2017-0186

**Published:** 2018

**Authors:** Bruno Pereira Valdigem, Dalmo A. R. Moreira, Rogerio B. Andalaft, Maria Virginia Tavares Santana, Carlos Anibal Sierra-Reyes, Carolina Mizzacci

**Affiliations:** 1 Instituto Dante Pazzanese de Cardiologia, São Paulo, Brazil.

**Keywords:** Arrhythmias, Cardiac, Tetralogy of Fallot, Heart Defects, Congenital, Catheter Ablation

## Abstract

Tetralogy of Fallot (ToF) is one of the most prevalent congenital heart disease. Its surgical corrections may haemodinamically correct a disease, but the incisions may create scars that will originate ventricular arrhythmias. Even though life threatening arrhythmias are not common, some patients present unstable ventricular tachycardia (VT) of ectopic ventricular beats triggering heart failure and symptoms. We describe the treatment of a 16-years-old woman with late ToF repair and drug refractory Implantable cardioverter defibrillator (ICD) shocks. The patient underwent successful ablation of VT using X-ray and anatomic landmarks without the use of electroanatomical mapping. We were able to reduce drugs after one month of ablation and improve quality of life and symptoms. In this paper we describe the indications and perform a brief review of the key points for successful radiofrequency catheter ablation of VT in ToF patients.

**Table:** 

Abbreviations, acronyms & symbols	
CHD	= Congenital heart disease
EA	= Electroanatomical
EGM	= Electrogram
EKG	= Electrocardiography
ICD	= Implantable cardioverter defibrillator
RV	= Right ventricle
RVOT	= Right ventricular outflow tract
TEB	= Tecnologia Eletronica Brasileira
ToF	= Tetralogy of Fallot
VSDC	= Ventricular septal defect closure
VT	= Ventricular tachycardia

## INTRODUCTION

Tetralogy of Fallot (ToF) is a congenital heart disease (CHD) that can present with a great spectrum of severity and anatomy. The surgical corrections are mostly guided by severity of pulmonary flow restriction and right ventricle (RV) hemodynamic compromise. Ventricular arrhythmias are common, even though clinically relevant ventricular tachycardia (VT) is a rare feature, and it is usually related to ominous prognosis. Most ventricular arrhythmias are a marker of prosthesis dysfunction or need for another open heart procedure. In some cases, though, VT can arise from scar related reentry circuits created by the previous surgical procedures. Amiodarone and beta-blockers can diminish arrhythmia burden, but are often less than effective. Ablation using electroanatomical mapping has been widely described by previous authors and the same authors have described the four main VT circuits that comprise most of the ventricular arrhythmias in this population. Even though electroanatomical (EA) mapping is available in many countries, some developing hospitals are not entitled to this technology. In this report we describe one alternative approach of VT ablation in late ToF repair using X-ray and anatomical landmarks, without aid of EA mapping.

## CASE REPORT

We present the case of a 16-year-old ToF patient, born in 2000. On a ventricular septal defect of 7.5 mm, the first surgical procedure was performed in 2001 (pulmonary commissurotomy + closure of ventricular septal defect + infundibulectomy), the second procedure in 2008 (homograft) and the third procedure was performed in 2009 due to homograft dysfunction, calcification and regurgitation [implantation of pulmonary valve bioprosthesis number 25 + and surgical ablation of ventricular ectopic beats arising from right ventricular outflow tract (RVOT), aiming for ventricular ectopy reduction using surgical mapping and electrocautery ablation]. The patient presented severe scoliosis, but was otherwise physically active.

When the patient was 14 years old she presented with palpitations and dizziness. Rest electrocardiography (EKG) showed a sinus rhythm of 67 bpm and right bundle branch block. Holter recordings at that time recorded 295 ventricular tachycardias, the longest with 107 beats (129 bpm) and the fastest lasting with 15 beats (136 bpm).

A single chamber implantable cardioverter defibrillator (ICD) was implanted in November of 2015, and amiodarone and metoprolol were initiated and the dosage uptitrated to amiodarone 600 mg/day, metoprolol 200 mg/day and phenytoin 200 mg/day. Oral magnesium was added to the treatment regimen without adequate response.

The patient presented 155 new VT episodes since the implant, 18 of which elicited therapy (total of 23 shocks). Last admission for appropriate shock was in October 2016, when ablation attempt was programmed. Echocardiogram at the time referred to moderate RV enlargement (evaluated in a subjective two-observer analysis including at least one CHD specialist, as protocol in our institution) with normal RV and left ventricular ejection fraction, ventricular patch and pulmonary prosthesis with normal function (pulmonary arterial pressure of 40 mmHg) and mild tricuspid regurgitation. Between shocks, the patient showed mild heart failure symptoms (fatigue during moderate activities).

**Fig. 1:**
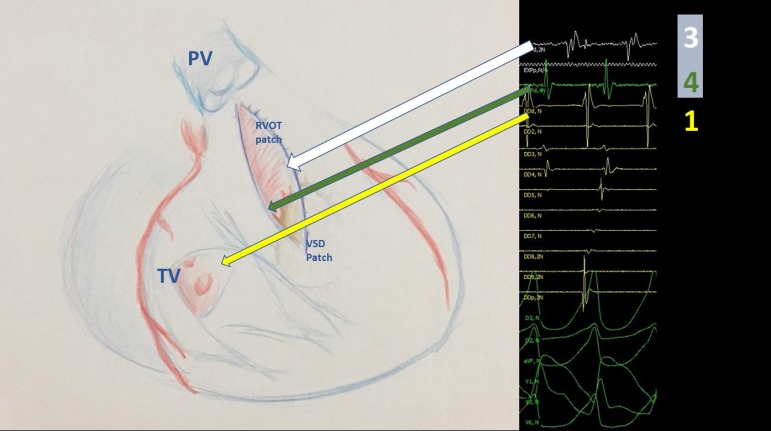
Disposition of catheters during EPS in search of mid-diastolic potentials. Proximal 20 - pole deflectable catheter - in yellow - on isthmus number 4 (between septal tricuspid valve and VSD patch. Ablation catheter - white line - on isthmus number 3 (between pulmonary valve and VSD patch), Quadripolar catheter - green line - on isthmus number 1 (between infundibulectomy and lateral tricuspid valve). There was no intention in mapping possible isthmus number 2 because the first surgery was performed along the pulmonary valve, thus surgically connecting the infundibulectomy patch with the pulmonary artery and then replacing it for the pulmonary prosthesis. PV = pulmonary valve; RVOT = Right ventricular outflow tract; TV = tricuspid valve; VSD = ventricular septal defect.

Ablation was performed under general anesthesia, fluoroscopy (GE 9900, General Electrics, USA), and Tecnologia Eletronica Brasileira (TEB) recording systems for electrical signal acquisition and stimulation. The pulmonary valve was identified using a pig tail 5F catheter and iodine contrast to locate the pulmonary prosthesis. A 20-pole catheter was located from septal wall (proximal end), along the anterior portion of the RVOT, to the RV free wall (distal end). The disposition of the catheters was diagramed as showed in Figure 1.

**Fig. 2:**
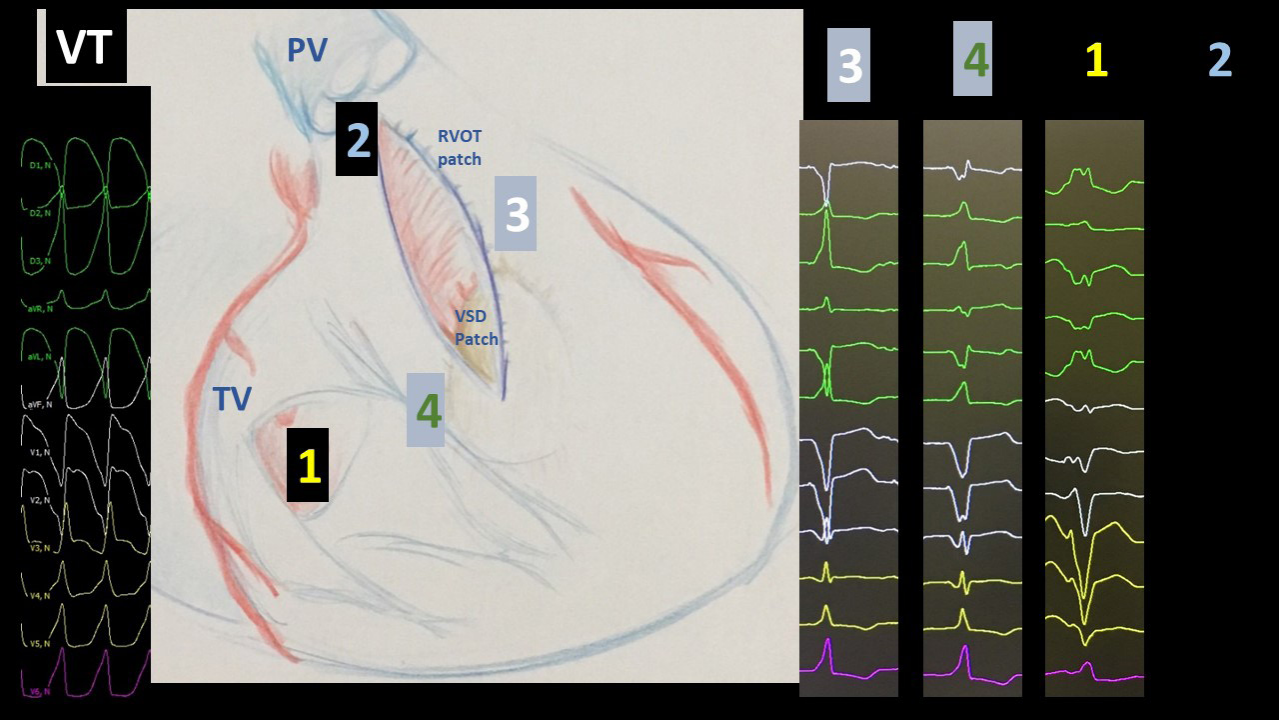
During EPS with S1 600 ms and S2 340 ms unstable VT was repeatedly induced and the cycle length was equal to the ICD recordings. The mid-diastolic EGM from the ablation catheter positioned between the VSDC and the pulmonary prosthesis was considered significant and pacing from that site with 500 ms cycle length was similar to the induced VT (site three). Previously, ablation pace mapping from the ablation sites were tried for mapping most probable isthmus. We can see pacing from isthmus 3 (between VSD patch and pulmonary valve), isthmus 4 (between VSD patch and tricuspid valve) and isthmus 1 (between RVOT patch and tricuspid valve). Isthmus 2 was not considered for ablation since the scar of the first surgery connected the RVOT and the pulmonary artery and no local EGM was found in this area to be treated. PV = pulmonary valve; RVOT = Right ventricular outflow tract; TV = tricuspid valve; VSD = ventricular septal defect; VT = Ventricular tachycardia.

Using that catheter, we were able to identify the landmarks that would be used to perform the ablation lines. The septal patch and the RVOT patch were identified by the low voltage areas (and non-capture using high output pacing), meaning absence of viable muscle. Before ablation, catheters were positioned on each of the four most frequent isthmus. Since the first surgery connected the RVOT patch and the pulmonary valve, and no electrical activity was found in isthmus 2, we assumed the surgical procedures performed earlier deemed mapping of this area unnecessary. Electrical programmed stimulation was performed using up to three extra stimuli and ventricular burst, which started an unstable VT. The VT was interrupted with external electrical cardioversion. Pacing was performed along isthmus 1, 3 and 4 (Figure 2). Mid-diastolic potentials were found during VT on isthmus 3. Pacing from that site was also similar to the induced VT.

**Fig. 3:**
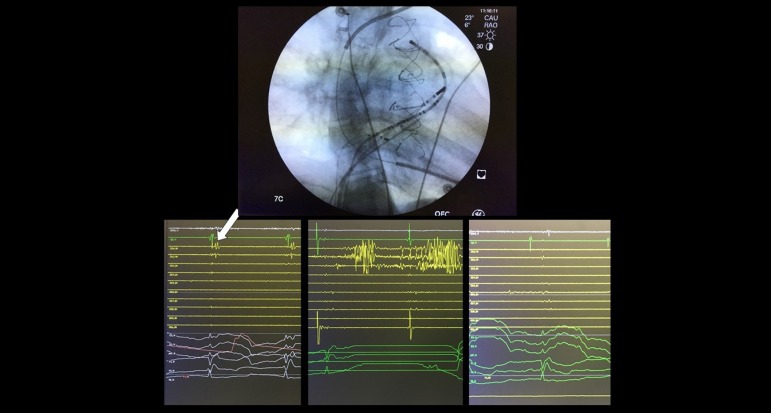
Ablation lines between landmarks successfully interrupting VT circuit (On fluoroscopy we can see a 20-pole deflectable catheter along isthmus number 3. A pig tail catheter was used to mark the pulmonary prosthesis. On the lower panels ablation of late fragmented late potentials (arrow) along the 20-pole deflectable catheter between the VSD patch and pulmonary prosthesis (lower left before ablation, middle during ablation and right lower after completion of the line). Other radiofrequency delivery was performed in order to extinguish any late potentials observed along that line.

**Fig. 4:**
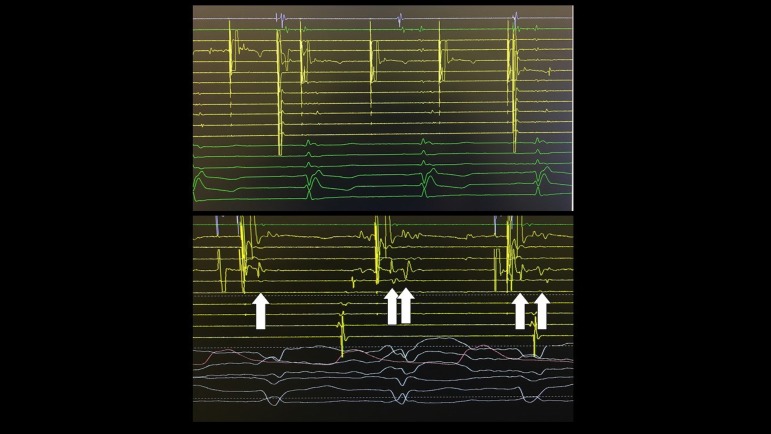
Upper panel: pacing along the line after ablation did not capture ventricular myocardium. Lower panel: double ventricular potential increase during pacing from one side of the line during ablation (arrows).

Ablation was performed from the bioprosthesis (identified using a pig tail catheter and pulmonary angiogram, as well as absence of electrical activity) to the VSDC (also, absence of electrical activity). We decided to create an ablation line between the pulmonary prosthesis and the VSDC using an 8 mm tip catheter and 70W in 60 degrees Celsius for 90 seconds in each site. The 20-pole deflectable was positioned along the line, and ventricular double potentials or electrical non-capture along the line were sought as interruption criteria (Figure 3). A roving 20-pole deflectable catheter was used to record the absence of electrical activity after ablation and to allow the operator to create an imaginary line of ablation along the catheter. After ablation pacing from one side, the ablation was performed in order to register the absence of electrical signal along the line and increased time from stimulus to local EGM from one side of the line to another (Figure 4). That allowed us to infer blockage between the two anatomical landmarks.

After successful ablation of this isthmus we performed three additional lines using a similar method on the three remaining isthmus. After ablation, new attempts to induce the VT, with or without drug elicitation were unsuccessful (new protocol of S1 450 ms, S2 300 ms, S3 220 ms and S4 200 ms and ventricular burst using a 220 ms cycle in the right ventricular apex and RVOT).

The patient was on amiodarone 200 mg with metoprolol 100 mg a day for twelve months and no VT episode, sustained or not, was observed on ICD or Holter recordings. The patient showed improvement of heart failure symptoms. The drug reduction was 66% for amiodarone, 50% for metoprolol and interruption of phenytoin and magnesium.

## DISCUSSION

As of 2001, there were estimated to be 800,000 adults living with CHD in the United States alone^[1]^. The Brazilian database suggests an incidence of close to 25,000 newborns with CHD in 2010, and among those 973 were born with the diagnosis of ToF^[2]^. In a recent survey published in Brazilian Journal of Cardiovascular Surgery, close to 1.6% of the heart surgeries performed in Brazil are on congenital heart patients^[3]^. ToF is one of the most frequent surgically corrected CHD present in our country, as it afflicts nearly 10% of all CHD patients. Even though the prognosis of ToF patients has improved, late ventricular arrhythmias remain a risk during the long term. VT can be found in near 11.9% of the cases, and sudden cardiac death takes a toll in up to 8.3% of patients after 35 years of follow up^[4]^.

First line therapy for asymptomatic patients with ToF and non-sustained ventricular arrhythmias remain beta blockers, especially in those with preserved ventricular function^[3]^. Patients with syncope and/or sustained VT require invasive evaluation, as well as assessment of ventricular function and prosthesis dysfunction. In the event of need for another surgical procedure, invasive electrical programmed stimulation can guide surgical ablation and identify the culprit isthmus^[5]^.

In patients who do not need further surgical procedure, catheter ablation should be considered an option. The indications of ICD in this set of patients remain despite the procedure, and in patients who are already ICD recipients, ablation could be a reasonable alternative to high dosage of amiodarone for long periods (most patients are in their mid 30s and should have a reasonable survival). As for patients in higher risk of sudden cardiac death that are candidates for another cardiac surgery, they should be carefully evaluated for indications of ICD, since the correction of hemodynamic problems alone does not correct arrhythmias in the long run^[6,7]^.

In 2007, Zeppenfeld et al.^[8]^ published a pivotal paper that evaluated electroanatomic mapping of 11 patients with sustained VT and late surgically corrected ToF. The paper evolved to the largest series of VT ablation of 74 patients with surgically corrected ToF (authored by Kapel et al.^[9]^, in 2017). The series described four locations of VT circuits and related them to anatomical landmarks. The most usual circuit was related to one of the four isthmus in 37 of the 41 patients in which VT was induced. Out of 71 patients, only 2 presented VT unrelated to one of the four isthmus.

The most frequent isthmus (27 VTs) was present between the pulmonary valve/prosthesis and the VSDC (Isthmus 3), the second (10 VTs) was between the tricuspid valve and the RVOT incision/patch (Isthmus 1), then (2 VT) present between the pulmonary valve/prosthesis and the RVOT incision/patch (Isthmus 2) and the last VT between the VSDC and the tricuspid valve (Isthmus 4). Notably, the narrowest isthmus presented the slowest electrical conduction and the most probable VT circuit. VT ablation was performed in slow conducting isthmus in 63 patients (out of 74) and they all remained uneventful after a medium follow up of 55 months^[9]^.

The lack of EA mapping in public health in Brazil is one of the major setbacks for electrophysiology. Patients with structural heart disease and the need for interventional procedures are often subject to toxic antiarrhythmic drug dosage and scarcely studied drug combinations^[10]^. ICD batteries are frequently depleted earlier than expected due to shocks that might be avoided, causing intense emotional and physical extenuation. Cases like the one reported are referred to heart transplant more frequently than to VT ablation in most centers.

We present a successful approach for VT ablation in this setting of structural heart disease without EA mapping. The possibility of a successful ablation was enhanced by knowledge of the surgical procedure performed and lengthy discussion with the cardiac surgeons and chat reviews. Since the anatomical landmarks are electrically non-excitable tissue (*i.e.* bovine pericardial patch and tricuspid valve), pacing on the edge of the landmark was used to identify the end of the myocardium and the beginning of the surgical repair. Adequate positioning of catheters on each isthmus prior to VT induction allowed us to identify mid-diastolic potentials and probable isthmus, and an increased probability was observed after contrasting the 12-lead EKG obtained during pacing from that site and the 12-lead of the induced VT.

The use of long multipolar catheters to guide the ablation line was extremely helpful, both as a form of guiding the ablation catheter and as a way of identifying the abolition of EGM along this same line.

The authors believe that this step by step procedure will help electrophysiologists enhance VT control in ToF patients when substrate ablation using EA mapping is not an option.

## CONCLUSION

VT ablation in patients with ToF is feasible and should be considered when there is no hemodynamic anatomical compromise or prosthetic failure. Ablation is most of the times related to four critical isthmus, and even though EA mapping is a potentially beneficial instrument for increasing ablation efficacy, conventional mapping and ablation should be performed and can elicit benefits in reducing drugs and ICD therapies.

**Table:** 

Authors' roles & responsibilities	
BPV	Substantial contributions to the conception or design of the work; or the acquisition, analysis, or interpretation of data for the work; final approval of the version to be published
DARM	Substantial contributions to the conception or design of the work; or the acquisition, analysis, or interpretation of data for the work; final approval of the version to be published
RBA	Substantial contributions to the conception or design of the work; or the acquisition, analysis, or interpretation of data for the work; final approval of the version to be published
MVTS	Substantial contributions to the conception or design of the work; or the acquisition, analysis, or interpretation of data for the work; final approval of the version to be published
CASR	Substantial contributions to the conception or design of the work; or the acquisition, analysis, or interpretation of data for the work; final approval of the version to be published
CM	Substantial contributions to the conception or design of the work; or the acquisition, analysis, or interpretation of data for the work; final approval of the version to be published
